# Multicentric Castleman disease of hyaline vascular variant presenting with unusual systemic manifestations: a case report

**DOI:** 10.1186/s13256-017-1294-3

**Published:** 2017-05-14

**Authors:** B. M. D. B. Basnayake, A. W. M. Wazil, T. Kannangara, N. V. I. Ratnatunga, S. Hewamana, A. M. Ameer

**Affiliations:** 1 Department of Medicine, Teaching Hospital Kandy, Kandy, Sri Lanka; 2 Department of Nephrology, Teaching Hospital Kandy, Kandy, Sri Lanka; 3 Department of Pathology, Faculty of Medicine, University of Peradeniya, Peradeniya, Sri Lanka; 4 Department of Hematology and Hemato-oncology, Lanka Hospitals, Colombo, Sri Lanka

**Keywords:** Castleman disease, Hyaline-vascular, Multicentric, Sri Lanka, Multiple system involvement, Rituximab, Case report

## Abstract

**Background:**

Castleman disease is a rare lymphoproliferative disorder presenting with localized or disseminated lymphadenopathy and systemic manifestations. It can be categorized in numerous ways, such as unicentric versus multicentric, histopathological variants (hyaline-vascular, plasma cell, and mixed), or subtypes based on causative viral infections (human immunodeficiency virus, human herpesvirus-8, or Kaposi sarcoma herpesvirus). Presentation ranges from asymptomatic to symptoms involving multiple organs. Even though the exact mechanism of pathogenesis is unknown, treatment is directed toward possible etiologies such as interleukin-6, cluster of differentiation 20, and viral agents.

**Case presentation:**

A 36-year-old Sri Lankan woman presented with generalized body swelling and foamy urine of 2 weeks’ duration. Examination revealed pallor; generalized edema; axillary, cervical, and inguinal lymphadenopathy; hypertension; and hepatomegaly. Investigations showed bicytopenia, nephrotic range proteinuria with hypoalbuminemia, hypogammaglobulinemia, and features of hyaline-vascular type Castleman disease in a lymph node biopsy. She was managed with rituximab and had good clinical improvement.

**Conclusions:**

Castleman disease has a broad spectrum of clinical manifestations, disease pathogeneses, and associations and/or complications. Medical professionals need to be familiar with this spectrum because timely diagnosis and aggressive targeted therapy are the cornerstones of managing these patients.

## Background

Castleman disease (CD), also referred to as *angiofollicular lymph node hyperplasia* or *giant lymph node hyperplasia*, is a nonclonal lymphoproliferative disorder affecting single or generalized lymph node stations [1, 2]. However, it has the potential to affect any organ and thus presents with diverse systemic manifestations. CD has three major histopathological variants, namely hyaline-vascular type, plasma cell type, and the mixed type [3]. According to the disease dissemination, CD is additionally categorized into unicentric or localized form and multicentric or disseminated systemic form [2–4]. The hyaline-vascular variant, which accounts for 90% of cases, is characterized by capillary proliferation with small hyaline-vascular follicles and has a benign clinical course. The unicentric form is common in this variant, and though it is usually asymptomatic, presentation with a mass lesion is possible. In contrast, the plasma cell variant accounts for less than 10% of cases, and the multicentric form is the most common presentation. It is frequently associated with systemic manifestations such as constitutional symptoms (fever, night sweats, and malaise), hepatosplenomegaly, marked lymphadenopathy, and hematological (commonly anemia or thrombocytopenia) and/or immunological abnormalities [2, 3, 5]. We report a case of a Sri Lankan patient with the multicentric hyaline-vascular variant of CD who presented with unusual systemic manifestations, namely nephrotic range proteinuria, marked lymphadenopathy, hepatomegaly, and hematological abnormalities.

## Case presentation

A 36-year-old, previously healthy Sri Lankan woman presented to our tertiary care hospital with generalized body swelling and foamy urine of 2 weeks’ duration. Her physical examination revealed that she was pale and edematous. She had bilateral axillary lymph node enlargement that was nontender (right 3 × 2 cm, left 1 × 2 cm), as well as multiple cervical and inguinal lymph nodes measuring less than 1 cm. Her blood pressure was 170/90 mmHg. She had nontender hepatomegaly 7 cm below the right costal margin. Laboratory investigations revealed a hemoglobin level of 9.5 g/dl, a platelet count of 52 × 10^9^/L, and a white blood cell count 12.8 × 10^9^. Her reticulocyte count was 1.2%. Blood examination revealed normochromic normocytic red cells, absolute neutrophil leukocytosis, and large platelets with moderate thrombocytopenia.

The appearance of her bone marrow biopsy was compatible with granulocytic proliferation with normal megakaryopoiesis, indicating an inflammatory process. Initially, her serum creatinine level was 1.08 mg/dl, but it rose within 1 week to 3.2 mg/dl. Her serum electrolytes were normal. Her aspartate aminotransferase level was 15 U/L, and her alanine aminotransferase level was 29.7 U/L. Her total protein was 5.8 g/dl with albumin 2.7 g/dl. Her international normalized ratio was 1.09. Her alkaline phosphatase level was 467.8 IU/L. A urine full report showed pus cells 8–10 and red cells 0–1 in high power field, and a highly positive result for albumin. Her urine protein excretion was high at 3.17 g (normal range 0–0.15 g) for 24 hours. Her urine protein creatinine ratio was 3868 mg/g of creatinine (normal range <150 mg/g of creatinine).

An ultrasound scan of the patient’s abdomen showed hepatomegaly of 16.8 cm in size without focal lesions, moderate ascites, and normal-sized kidneys. She had no splenomegaly. A chest x-ray showed bilateral mild pleural effusion. The results of two-dimensional echocardiography were normal. Her lipid profile was normal. Her C-reactive protein level was 49 mg/dl. Her erythrocyte sedimentation rate was 34 mm in the first hour. Results of blood and urine cultures were negative. Her human immunodeficiency virus 1 (HIV-1) and HIV-2 antibodies, antibody to *Mycoplasma pneumoniae*, hepatitis B surface antigen, and hepatitis C antibody test results were negative. Antinuclear antibodies, double-stranded deoxyribonucleic acid (DNA), and direct Coombs test results were negative. Serum protein electrophoresis revealed features of an acute-phase reaction with hypogammaglobulinemia.

**Fig. 1:**
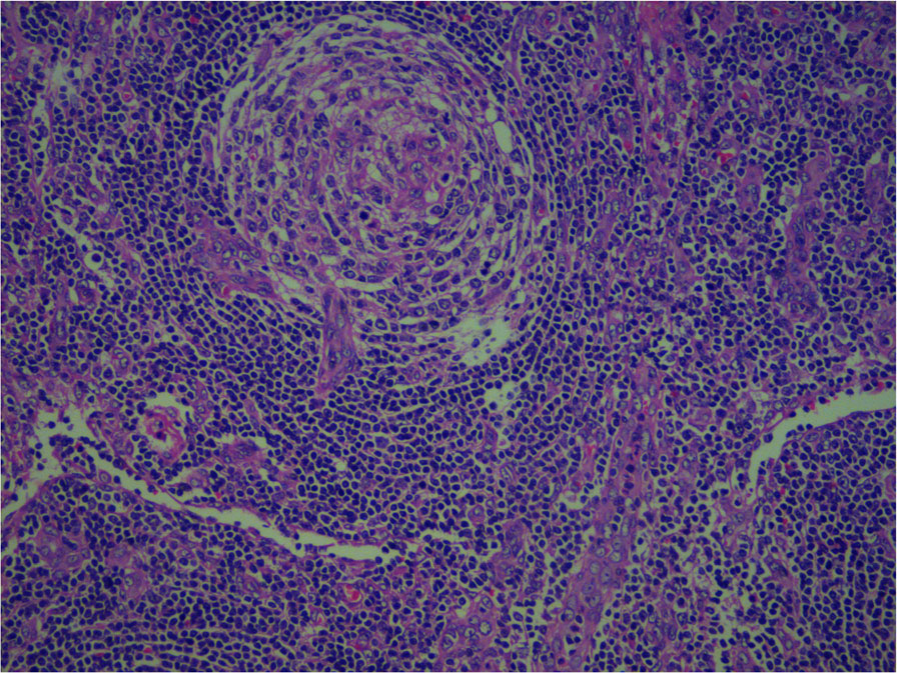
Lymph node biopsy showing features of Castleman disease of hyaline-vascular type. Numerous small follicular structures, follicular dendritic type cells, and blood vessels, some with hyaline walls in the centers of the follicles, can be seen. Concentric layers of lymphocytes around the follicles corresponding to the mantle zone, as well as small lymphocytes among the venules with occasional reactive follicles, are also shown

An axillary lymph node biopsy (Fig. 1) revealed numerous small follicular structures. The centers of these follicles were small and consisted of follicular dendritic type cells resembling burnt-out follicles. Blood vessels, some with hyaline walls, were seen in the centers of the follicles. Concentric layers of lymphocytes were seen around the follicles corresponding to the mantle zone. The parafollicular tissue also showed extensive proliferation of the postcapillary venules. Small lymphocytes were seen among the venules. Occasional reactive follicles were seen. The appearance was highly suggestive of CD of hyaline vascular type with lymphoid subtype.

A diagnosis of multicentric CD of hyaline-vascular variant was made. The patient was started on diuretics and antihypertensives and commenced on rituximab 375 mg/m^2^ weekly for 4 weeks. She experienced dramatic clinical, biochemical, and hematological improvement. After a 7-week hospital stay, she was discharged. One month after discharge, her laboratory parameters had normalized, and she was clinically well. She was followed at the nephrology clinic, and no complications were encountered.

## Discussion

CD first appeared in the medical literature in 1956 with 13 cases of localized mediastinal lymph node hyperplasia described by Dr. Benjamin Castleman. He originally described the classical histopathological features of the hyaline-vascular variant and unicentric distribution of the disease [6]. However, CD can affect multiple regions of the body, mainly lymphatic tissue (70% in the chest, 15% in the neck, 15% in the abdomen and pelvis) and extralymphatic sites such as the lungs, liver, spleen, larynx, kidneys, parotid glands, pancreas, meninges, and muscles [1, 7]. Thus, the clinical presentation has a wide variation and may well mimic multiple disease entities. Our patient was initially investigated for hematological malignancies with a main focus on lymphomas because she had marked lymphadenopathy, hepatomegaly, and hematological abnormalities.

Hyaline-vascular CD usually occurs in young adults with no gender predilection, and about 90% of cases are unicentric [1, 2]. Rarely, the multicentric form may occur as in our patient with axillary, cervical, and inguinal lymph node involvement. In the multicentric form, there is aggressive disease progression, and it may be associated with conditions such as POEMS syndrome (polyneuropathy, organomegaly, endocrinopathy, multiple myeloma, and skin changes), amyloidosis, Kaposi sarcoma, renal insufficiency, and HIV and human herpesvirus-8 infections (Table 1) [1, 2, 5].

**Table 1:** Comparison of Castleman disease subtypes

	Hyaline-vascular CD	Plasma cell CD
Presentation	Unicentric more common than multicentric	Multicentric more common than unicentric
Distribution of lymphadenopathy	Central (mediastinal, cervical) most common	Central and peripheral lymph nodes
Age	Third and fourth decades of life	Unicentric form in young adults Multicentric form in elderly (median sixth decade of life)
Prevalence	Common	Less common
Etiology and pathology	Unknown; possibly follicular dendritic cell abnormalities	IL-6, possibly plasma cell abnormalities, infection with HHV-8
Symptoms	Incidental or occasional constitutional symptoms	Frequent constitutional symptoms and hematological abnormalities
Clinical course	Benign	Usually aggressive
Organomegaly	Rare	Frequent (hepatosplenomegaly)
Associated lesions	Paraneoplastic pemphigus, thrombotic thrombocytopenic purpura, plasmacytoid dendritic cell collections	POEMS syndrome, HIV infection, Kaposi sarcoma, amyloidosis, renal insufficiency
Progression to lymphoma	Rare	Common
Treatment	Surgical resection, radiotherapy if inoperable	Chemotherapy, antiviral therapy

*Abbreviations: CD* Castleman disease, *HHV* Human herpesvirus, *IL* Interleukin, *POEMS* Polyneuropathy, organomegaly, endocrinopathy, multiple myeloma, and skin changes, *HIV* Human immunodeficiency virus

Renal manifestations of CD are uncommon, and pathologies include amyloidosis, minimal change disease, mesangial proliferative glomerulonephritis, membranous glomerulonephritis, and interstitial nephritis. Patients with renal involvement can develop albuminuria, proteinuria, hematuria, hypertension, and chronic renal failure [8, 9]. Renal biopsy, which would have provided more information on our patient’s renal pathology, was not done because she had low platelets. Suggested potential etiological factors for CD are lymphoid-hamartomatous hyperplasia, autoimmune phenomena, immunodeficiency, chronic low-grade inflammation, and excess production of interleukin-6 (IL-6). In the hyaline-vascular variant, follicular dendritic cell abnormalities and vascular endothelial growth factor have been demonstrated as the causative agents [5, 10].

Treatment of CD is directed at the suggested disease pathogenesis. Surgical excision is used for unicentric disease of either of the hyaline-vascular or plasma cell variant, but it is rarely used for the multicentric form. Cytoreductive therapy (chemotherapy) can be used for multicentric CD. The most common chemotherapeutic regimens used are cyclophosphamide, vincristine, doxorubicin, and either prednisone (“CHOP” therapy) or dexamethasone (“CVAD” therapy) [2]. Some studies show benefits in radiation therapy and in immune modulators such as steroids, interferon-α, all-*trans* retinoic acid, and thalidomide in the management of CD [2, 11, 12]. Other treatment options are monoclonal antibodies, which include anti-IL-6 monoclonal antibody (altizumab) and cluster of differentiation-20 monoclonal antibody (rituximab). In our patient, rituximab proved to be a successful treatment modality. Several antiviral agents, such as ganciclovir, foscarnet, cidofovir, and valganciclovir, are used in the management of viremia-associated CD [2, 11–15].

## Conclusions

Although hyaline-vascular CD usually has a benign clinical course, our patient presented with multicentric distribution and multisystemic involvement. CD needs to be considered in the differential diagnosis when a patient presents with generalized lymphadenopathy and systemic manifestations.

## Abbreviations

CD: Castleman disease
HHV: Human herpesvirus
IL: Interleukin
POEMS: Polyneuropathy, organomegaly, endocrinopathy, multiple myeloma, and skin changes
